# Perception About Factors Affecting Patient Adherence With Cardiac Medicines: A Cross‐Sectional Study

**DOI:** 10.1002/hsr2.70532

**Published:** 2025-03-05

**Authors:** Jesmin Sultana, Rumman Reza, Mohammad Saifuddin, Zakir Hossain Bhuiyan

**Affiliations:** ^1^ Department of Business Administration University of Asia Pacific Dhaka Bangladesh; ^2^ Department of Pharmacy University of Dhaka Dhaka Bangladesh; ^3^ Department of Mathematics and Statistics Bangladesh University of Business and Technology Mirpur Bangladesh; ^4^ Department of Marketing University of Dhaka Dhaka Bangladesh

**Keywords:** cardiac medicine, cardiovascular disease, drug adherence, factor analysis, patient compliance

## Abstract

**Background and Aims:**

Long‐term medicines are frequently recommended to cardiovascular patients to control hypertension and lower heart burden. Patient adherence to cardiovascular medicine is crucial since stopping it increases the risk of cardiac adverse events and poses a bigger risk to health. Perception of manufacturers, prescribers and patients were taken into account to identify the most important variables that can have a positive impact on patient satisfaction and thereby increase adherence to prescribed cardiovascular drug therapy.

**Methods:**

The empirical study was conducted using quantitative survey method. Questionnaire contained variables influencing patient compliance measured by a 5‐point Likert scale where 1 is *strongly disagree* and 5 *strongly agree*. Questions were handed over by drop‐off and collect method. This study was conducted in 15 tertiary health‐care service centers and 18 pharmaceutical companies across Dhaka district, Bangladesh. A total of 156 cardiologists, 200 cardiac patients and 180 marketing professionals (manufacturers) were included in the survey.

**Results:**

A total of 11 variables that can affect patient compliance were utilized to create correlation matrix and subsequently to carry on exploratory factor analysis. Hypothesis testing was done by using the regression model. Forty‐eight percent of respondents strongly agreed that efficacy of cardiac medicine influences patient compliance. Most of the stakeholders (69%) agreed that the company reputation of cardiac medicine suppliers is an important criterion. Availability and affordability were highly positively correlated variables. The variables were grouped under four categories according to factor loading scores. The four factors were medicinal issues of product strategies, economic issues of price strategies, convenience issues of place strategies and communication issues of promotional strategies.

**Conclusion:**

The findings of this study contribute to the comprehension of the factors influencing patient compliance from multiple dimensions. Policy makers should implement and promote policies that encourage patient adherence to long‐term prescription drugs.

## 1 Introduction

An estimated 17.9 million people die from cardiovascular diseases (CVDs) annually, which are the main cause of death worldwide [1]. Cardiovascular patients are often prescribed long‐term medications to manage hypertension and decrease heart load [2]. Patient compliance with cardiovascular medication is important as discontinuation pose greater risk to health and increase likelihood of cardiac adverse events [3]. Patient compliance, also known as adherence, is the degree or extent of adherence to the provider's daily treatment recommendations with regard to timing, dosage, and frequency [4]. It can be characterized as “the extent to which a patient act in accordance with the prescribed interval, and dose of a dosing regimen“ [5]. For chronic diseases where lifestyle management does not suffice and medication intervention is necessary, the significance of patient adherence to recommended treatment regimen by physician is paramount [6]. Only when a patient obtains and takes the appropriate medication to treat the symptom or disease, in the appropriate formulation and dose, at the correct interval, and for the proper duration, can medications treat diseases and lessen suffering [7].

There are many underlying factors that can influence a patient's medication consumption behavior. Variables such as availability, affordability and price of medicine may affect patient's satisfaction with the treatment regimen, which ultimately has positive or negative influence on drug compliance [8, 9, 10]. The efficacy of a drug can be an important issue when it comes to a patient's decision to further adhere to the prescription regimen or discontinue medication and sought different opinion from prescriber on the ailment under consideration [11]. Several issues that are connected to the convenience of medicine buying can also influence patient adherence to long‐term prescribed drugs [12].

Improper adherence to prescribed medication can pose a threat to the potential benefits of Universal Health Coverage (UHC) by endangering individuals, decreasing the efficacy of medications (if antimicrobial resistance develops), and endangering the financial security of healthcare systems. Inappropriate usage issues are complicated and require multifaceted, coordinated responses since they do not have a single root cause [13]. The UHC's recommendations center on tactics that encourage and support the use of high‐quality medications by patients, healthcare professionals, insurers, supply chain managers, and others (including the pharmaceutical sector) [14]. Strong institutions that can produce evidence and put into practice policies based on that evidence are essential [15]. Improvements in clinical, public health, economic, and ethical results will result from these initiatives.

Pharmaceutical companies manufacture medications as products for the healthcare of patients who are primary buyers [16]. Although OTC medications can be acquired by patients without a prescription, in Bangladesh, most individuals rely on medical advice before purchasing any medications. One feature of the pharmaceutical care process is achieving specific and positive patient outcomes through the best use of medications [15, 16]. The evaluation of the outcome, the development of the care plan, and the assessment of the patient are the three primary components of the care process [17]. Factors that affect patient compliance must be critically assessed and properly scrutinized to achieve the best quality health care management which can assure patient compliance and hence result in positive patient outcomes. In low‐middle‐income and middle‐income countries like Bangladesh, there are many scopes for improvement in the total healthcare management of chronic diseases such as cardiovascular disease. Research works until now have mainly focused on the prescription pattern of cardiac medicines from physicians' end. However, integrated studies that focus on the adherence of patients to cardiac medicines from a multi‐faceted dimension is lacking in the context of Bangladesh [18, 19]. In the present work, the perceptions of patients (primary consumers of medicines) and prescribers have been collected and analyzed. In the present study, manufacturers' perceptions of the variables affecting patient compliance have also been evaluated to achieve a greater analytical window.

## 2 Methodology

### 2.1 Study Design

Cross‐sectional study design was adopted for the present study. Data collection for this research was carried out by the survey method. To get accurate and complete information, questionnaire is the most formalized tool of the survey method, which is the appropriate approach to examine the research hypothesis. To pretest the questionnaires utilized in this investigation, a pilot study was conducted. The variables included in the questionnaire were selected from an extensive literature review based on similar studies performed in other countries [6, 20, 21].

### 2.2 Questionnaire Development

During the pilot study's survey, it was noted that participants appeared irritated when asked to respond to time‐consuming, sensitive, or irrelevant items. The stakeholders were asked about the statements which they found ambiguous and difficult to comprehend. Their responses were recorded in a semi‐structured interview with the researchers. The ambiguous statements were removed from the final questionnaire. The stakeholders were asked to evaluate the finalized self‐explanatory questionnaires containing variables/attributes of cardiac medicine influencing patient compliance by a 5‐point Likert scale ranging from 1 to 5 where 1 is *strongly disagree* and 5 *strongly agree*. With the help of questionnaires, the researcher has achieved the objectives and the factors that impact overall patient compliance. Perception of manufacturers from pharmaceutical companies, cardiologists, cardiac patients in the cardiac department of different hospitals were considered.

### 2.3 Target Population

Doctors (cardiologists), patients (cardiac patients), and manufacturers are the sample components of the cardiac market segment for the current study and have the information needed. Only adult patients (age > 18 years) were included in the current study. Cardiologists included in the research work were randomly selected from a list of hospitals that were included in the study. To obtain hospital addresses and phone numbers, the Bangladesh Cardiologists Association (BCA) and Bangladesh Medical Association (BMA) provided a list of the cardiologists who were randomly selected for participating in the survey. The list of cardiology departments in both public and private tertiary‐care hospitals were utilized for the purpose of gathering information from cardiac patients. To prepare a list of manufacturers to be surveyed, the Bangladesh Pharmaceutical Industries Association (BPIA) was used as the sampling frame for the population of professionals. Inclusion criteria for patients were that the patients receiving treatment for cardiac ailment were selected for the study. Doctors with specialization in the field of cardiology were included in the survey.

### 2.4 Data Collection

The hospital branches picked the doctors and gave out the surveys to protect the privacy of the doctors and the patients. Each branch received questionnaire envelopes where each envelope contained 20–30 questionnaires for cardiologists and 60–90 questionnaires for patients. The branches then dispatched the packages to the randomly chosen doctors.

The following criteria were used to choose 3–5 patients for each doctor. A questionnaire envelope was distributed to up to 3–5 patients on one working day during the study period who visited the doctor at a specific time slot during each office hour (9.0 a.m. to 5.0 p.m. BDT). The completed questionnaire was given back to us once each question had been answered. The patients completed the survey after getting a questionnaire and an explanation of the survey from their doctors. Each patient gave us their completed survey in person. The Ethics Committee examined and gave its approval to this project before it began. All prescribers and patients were informed of the ongoing research and each participant gave their consent to participate in the current research study.

Manufacturers of cardiac medicines are the professionals who are directly involved at different stages, starting from medicine production to procurement. Manufacturers from top ten pharmaceutical companies according to market share of cardiac medicines in Bangladesh were selected for the current study. Manufacturers were chosen by stratified random sampling without replacement, 10 from each organization, to generate a representative sample. Each corporation is conceptually considered a stratum. 180 marketing professionals in total have been chosen using stratified random sampling with equitable distribution based on market share of the pharmaceutical company. The survey described in this present work was carried out with ethical approval from the University of Dhaka's Faculty of Biological Sciences. The survey questionnaire for the study was authorized by Dhaka University's Ethical Clearance Committee. The cardiologists who took part in the survey received a letter from the Department of Marketing at the University of Dhaka. Informed consent was collected from each and every participant in the study.

### 2.5 Calculation of Sample Size

The total population consists of three sub‐populations. Thus, the total population (*N*) has been divided into three different portions. Mathematically *N* = *N*_1_ + *N*_2_ + *N*_3_, where *N*_1_ = Number of cardiologists, *N*_2_ = Number of cardiac patients and *N*_3_ = Number of manufacturers. The sample size of *Ni* for each portion (sub‐population) is determined by using the following formula with 95% confidence interval and 7% margin of error (where, *i* = 1, 2, 3) [22].

$$Ni=\frac{\frac{z\alpha /2\times P(1-P)}{e^{2}}}{1+\frac{\left(z\alpha /2\times P(i-p)\right)}{Nie^{2}}}$$

### 2.6 Questionnaire Development

The questionnaires included multiple choice questions, a 5‐point Likert scale, and demographic data on the respondents. Gender, age, educational background, and years of experience with cardiac medications made up the demographic data. To measure the impact of different variables on doctors', patients', and professionals' perception regarding factors influencing patient adherence, scale‐based surveys with checkboxes for each assertion of satisfaction were created. The scale used was 1 to 5, with 1 *denoting severely disagree*, 2 *denoting disagree*, 3 *denoting neither agree nor disagree* or *neutral*, 4 *denoting agree*, and 5 *denoting strongly agree*. The three standard questionnaires for the three categories of stakeholders namely manufacturers, physicians, and patients were created and given to the appropriate parties.

### 2.7 Pilot Study to Ensure Reliability of Developed Questionnaire

Pilot study was conducted before finalizing and dissipating the final version of the questionnaire. A smaller number of study participants than the actual sample size was invited for a pilot study. Preliminary version of the questionnaire was used in the pilot study and opinions regarding questionnaire comprehensibility, precision and effectiveness to carry the intended message to the participants were collected through face‐to‐face interview of the participants. Time required to fill‐up the pre‐testing questionnaire were recorded using stop watch and the number of questions to be incorporated in the final version of the questionnaire were decided accordingly. The number of pre‐testing participants were 15 doctors, 20 patients and 10 manufacturers. Their opinions were carefully scrutinized and utilized to build the final version of the questionnaire. Only the questions which produced consistent results were included in the final copy. The questions that produced erratic and inconsistent results and also had negative comments from participants were discarded form the final version.

### 2.8 Hypothesis Development

The qualities or factors of marketing mix strategies that can influence patient compliance were used to establish the study's hypothesis, which represents marketing strategies and techniques from a variety of literature. In the Bangladeshi pharmaceutical market, the effect of the relevant variables on patient satisfaction was assessed by considering the opinions of patients, prescribers and manufacturers of cardiac medicine. The four concerns taken into account for the cardiac market segment's marketing strategies and techniques are as follows: (i) Medicinal issues of product strategies (ii) Economic issues of price strategies (iii) Convenience issues of place strategies and (iv) Communication issues of promotional strategies.

### 2.9 Data Analysis

All statistical calculations were carried out utilizing IBM SPSS for Windows. First, the reliability and validity of the Likert scale directed towards the properties of variables affecting patient compliance were determined. Cronbach's *α* coefficient was used to determine the dependability of the results. The Spearman–Brown formula is used to determine the expected number of individuals for each group (patient, prescriber, and manufacturer) needed to achieve the desired levels of reliability.

In the second stage, exploratory factor analysis was carried out using patients' data. Kaiser‐Meyer‐Olkin measure of sampling adequacy was calculated before carrying on factor analysis. Furthermore, Barlett's test of sphericity and chi‐squared values were calculated to ensure the sample size of the patient was adequate for carrying out factor analysis.

### 2.10 Data Visualization

Graphs and correlograms were prepared using advanced options in Microsoft Excel. Visual graphics were prepared using a Microsoft software package. Statistical analyses were conducted in IBM Statistical Package for the Social Sciences (SPSS) 26. Statistical plots were directly retrieved from IBM SPSS.

## 3 Result

### 3.1 Demographic Profiles of Respondents

A total of 1000 questionnaires were handed out to respondents. Only 560 out of 1000 questionnaires had been returned. Only 536 of the 560 replies were deemed to be fully complete in all respects and hence qualified for study. Among the 536 respondents, 156 were cardiologists, 200 were cardiac patients, and 180 were manufacturers.

The majority of cardiologists (76.6%) of the 156 total are men, and the remaining 23.4% are women. The majority of cardiologists (prescribers) who took part in the poll fall under the age category of 30–40 years old with 44.9% of them being in this age range. On the other hand, the percentages for the age groups 41–50 and over 50 are, respectively, 37.2% and 10.3%. The academic backgrounds of the participating cardiologists were found to be 38.5% MBBS, 15.4% FCPS, 28.2% MD, and 17.9% D. CARD and others. Two hundred patients in all took part in the study, with 59.5% men and 4.5% women. The majority of cardiac patients who have heart disease are women. One hundred and eighty marketing professionals in all took part in the poll. 15% of them are women, while 85% of them are men. A larger proportion of manufacturers (marketing professionals) are male. 51.63% of the marketing professionals fall within the age group of 25–30 years. The demographic profiles of respondents are enlisted in Table 1.

**Table 1:** Demographic characteristics of patients, manufacturers, and prescribers who participated in the research survey.

Demographic characteristics	Percent(%) of manufacturers	Percent(%) of patients	Percent(%) of prescriber
Gender			
Male	85	59.5	76.6
Female	15	40.5	23.4
Age			
Below 25	2.78	3.5	0
25–30	51.63	2.5	7.7
31–35	31.11	7	10.8
36–40	10.56	10	34.1
40–50	2.68	28	37.2
Above 50	1.2	48	10.3
Education qualification of manufacturers			
MS. Pharm Tech	80	N/A	N/A
B. Pharm	14	N/A	N/A
MBBS, MBA, BSS, MPH	6.11	N/A	N/A
Education qualification of prescribers			
MS. Pharm Tech	N/A	N/A	80
B. Pharm	N/A	N/A	14
MBBS, MBA, BSS, MPH	N/A	N/A	6.11
Education qualification of patients			
Secondary School Certificate	N/A	13.5	N/A
Higher Secondary School Certificate	N/A	20	N/A
Honors	N/A	40.5	N/A
Others	N/A	26	N/A
Experience of selling cardiac medicine			
Below 5 years	68	N/A	N/A
5–10 years	25	N/A	N/A
11–15 years	4.4	N/A	N/A
Above 15 years	2.2	N/A	N/A
Experience as prescriber			
Less than 10	N/A	N/A	59
Above 10	N/A	N/A	41
Experience using cardiac medicine			
2–3 years	N/A	36.5	N/A
4–7 years	N/A	36.5	N/A
Above 8 years	N/A	27	N/A

### 3.2 Measurement of Reliability of Developed Questionnaire for Respondents

A group of survey items' internal consistency or reliability is measured by the Cronbach's *α* coefficient. If a group of items regularly assesses the same attribute, this statistic can be employed to assist in making that determination. On a standardized 0–1 scale, Cronbach's *α* assesses the degree of agreement. The same concept, trait, or construct is commonly the subject of many questions in surveys and assessment tools. A more detailed evaluation of the phenomenon can be developed by the test by having multiple items on the same aspect. A scale for the construct can be created by analysts by combining several relevant items. However, they must be certain that every item accurately measures the same concept before including different questions on a scale. That process is aided by Cronbach's *α* [23].

Reliability Testing indicates that the Cronbach's *α* of patients' data is 0.67 (number of items, *n* = 11) which can be considered to be within acceptable limit. The Cronbach's *α* for the questionnaires intended for prescribers is found at an acceptable level of 0.83 that indicates the variables of questionnaires are consistent. This implies that the questions were readily comprehensive and the responses that followed were not ambiguous.

### 3.3 Analysis of Impact Scores of Prescribers, Patients, and Manufacturers

**Figure 1:**
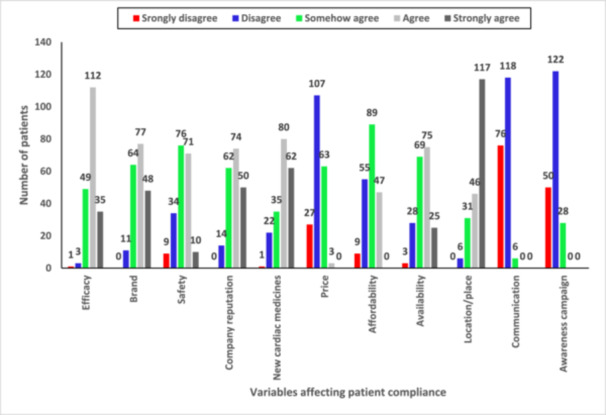
Bar plot showing number of patients under each category of strongly agree, disagree, somehow agree, agree and strongly agree with the statements about variables affecting patient adherence.

The most valued clients for medical professionals and pharmaceutical businesses are cardiac patients. Two hundred cardiac patients were asked to provide feedback on the characteristics of 11 components that influence compliance to treatment regimen. The number of patients giving their consent in either of the five categories of strongly disagree, disagree, neutral, agree, and strongly agree have been presented as a bar plot in Figure 1. Seventy‐one percent concurred that effectiveness and quality are well regarded. 59.6% concurred that doctors and patients are drawn to particular brands of cardiac medication. 39.7% of respondents agreed that pharmaceutical companies offer adequate information about the safety of the drug. 57% of respondents indicated their support for company's reputation. The notion that newer medications draw more users than older ones was rejected by 72.9%. 64.2% of cardiac patients disagreed that the cost of cardiac care does not correspond to its quality. 35.1% of respondents indicated they had neutral perspective on the cost of cardiac medications. 51% of cardiac patients agreed with the assertion that medications availability in retail pharmacies impact their adherence.

When asked whether location or distribution network strategies were important for retail pharmacies in Dhaka City, 75.1% of cardiac patients agreed. They were given the opportunity to voice their opinions regarding the claim that pharmaceutical manufacturing companies interact with them to learn about the effects of the medications. 91.4% of respondents disagreed that most of the companies interacted with them. The pharmaceutical industry's awareness effort to raise patient awareness of heart disease was discussed with the cardiac sufferers.

Ninety‐three percent physicians were in agreement that efficacy and quality were crucial for patient satisfaction while 6% disagreed. According to 69% of physicians, the brand name of cardiac medications matters more than their generic names. 77% of physicians stated that they are influenced by a company's reputation when recommending cardiac medications. In the context of new cardiac medicines, 21% of physicians disagreed that it has influence on patient adherence. 58% of cardiologists concur that cost reflects quality. 65% of doctors agreed that patient compliance can be enhanced when they take the market pricing of cardiac medications into account while prescribing.

79% of cardiologists believed that accessibility to medications is crucial. 59% of respondents agreed that the placement of a retail pharmacy that makes cardiac medications available everywhere in Dhaka City is crucial for doctors to prescribe the drugs, which has an impact on the companies' potential to increase sales.

Manufacturers are particularly concerned about the availability of medication at hospital and retail pharmacies because cardiac medication is essential and required on a daily basis. 50% of respondents said that a pharmacy's location affects sales. 29% of respondents were neutral, while 21% disagreed.

The impact of promotional strategies was seen among the many components of promotional mixed methods, according to the survey. Promotion has the greatest impact on consumer satisfaction, which in turn affects how and whether doctors recommend cardiac medications. A cardiac patient awareness campaign to avoid heart disease is essential for publicity and goodwill, according to 62% of respondents. Neutrality was discovered in 33% of them.

The assertion that company reputation truly impact doctors' prescriptions and sales of cardiac medication received favorable feedback from 76% of managers. The preference for newer cardiac medications over existing (older) medications was expressed by 41% of managers. Only 18% of marketing professionals disagreed with the assertion, while 41% were undecided.

**Figure 2:**
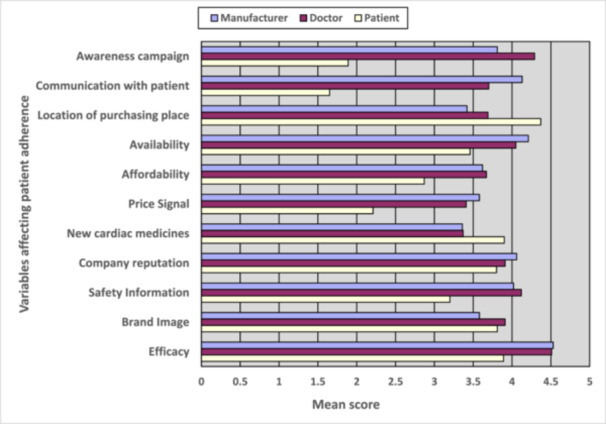
Bar plot showing comparison between mean scores of manufacturers, doctor and patient for the variables affecting patient adherence.

One‐third (36%) of the respondents agreed about the efficacy issue, while only 2% disagreed and 12% were undecided. On average, they gave it a 4.30 out of 5, with a standard deviation of 0.81. The mean scores of each type of respondents (manufacturer, patient, and doctor) has been presented in a bar plot in Figure 2. When it comes to the brand image of cardiac medicines, 25% of stakeholders strongly agreed, 35% agreed, 8% disagreed and the rest were neutral, with a mean score of 3.76. About 61% said that safety information about the cardiac medicines supplied by the companies affect patient satisfaction. Most of the stakeholders (69%) agreed that the company reputation of cardiac medicine suppliers is an important criterion, average score being 3.92. Around 21% of all the respondents strongly agreed and 31% agreed that new cardiac medicines have more impact on satisfaction than old ones. When it comes to the price of cardiac medicines, 36% disagreed that price is important for satisfaction. Most of the stakeholders (64.1%) said that the location of the pharmacy is imperative for their satisfaction, 25.9% were neutral and 9.1% disagreed, mean score being 3.85 (Figure 2).

### 3.4 Evaluation of Correlation Matrix of Variables Affecting Patient Adherence

**Figure 3:**
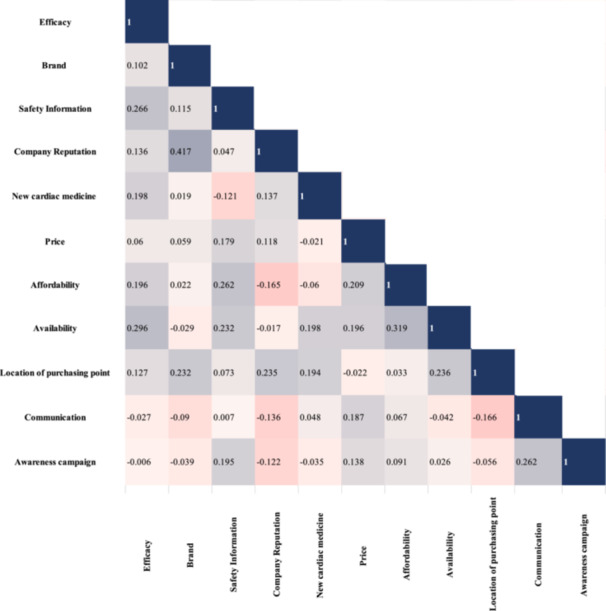
Correlation matrix displaying the correlation coefficients between the variables studied in this research.

A square matrix displaying the correlation coefficients between two variables is referred to as a correlation matrix. The strength and direction of the relationship between two variables in a straight line are determined by the correlation coefficient. In multivariate analysis and statistics, a correlation matrix is typically used to explore the relationships between several variables. To examine the relationships between each variable, a correlation matrix was developed. In this manner, it might be simpler for researchers to deal with problems in the future that are connected to one another and affect stakeholders' perceptions. The correlation coefficients of the variables are visualized in a correlation matrix given in Figure 3.

### 3.5 Sample Adequacy Measurement for Exploratory Factor Analysis

The Sample Adequacy test was carried out for Patients' data (*n* = 200). The sample is adequate for factor analysis as KMO value is more than 0.604 and significant value of Bartlett's test of Sphericity is good enough (df = 55) with approximate *χ* ^2^ value of 246.678. All these indicate the study sample can be utilized for factor analysis [24].

### 3.6 Exploratory Factor Analysis to Sort Variables Into Factors

**Figure 4:**
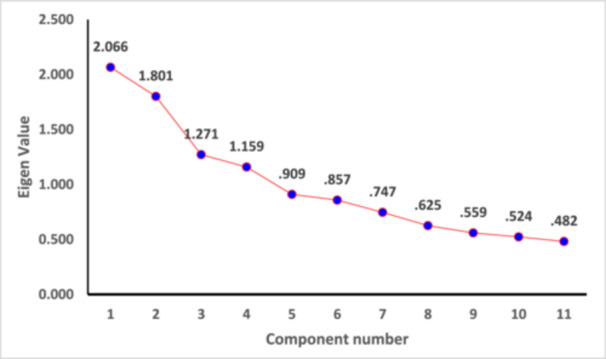
Scree plot of eigen values and variables components for patients' data.

The important issues are taken as basic factors of the research like medicinal benefit, economic benefit, convenience benefit and communication benefit. Relative important factors for patient adherence were identified by order of entry into Factor analysis for patients' data (*n* = 200). The four factors explained 57.246% of the total variance. The scree plot showed that the 11 variables can be effectively sorted into four factors. Availability, affordability, safety information, and efficacy were sorted under the category of Factor 1. Brand image, location of purchasing point, were sorted to Factor 2. Awareness campaign, price was categorized into Factor 3. New cardiac medicine was considered under Factor 4. Figure 4 shows the scree plot generated for the 11 variables studied in this research. The four factors were labelled as F1: Economic‐ Medicinal Benefit, F2: Medicinal‐Convenience Benefit, F3: Medicinal‐Communication Benefit, and F4: Medicinal benefit. The estimation of shared variance of the total variables is defined by communities. The factors that were retrieved account for the variable. The value of communality is predicted to be at least 0.40 for each variable. The communality values are depicted in Supporting Information S1: Table S1. Extracted Communalities from Extraction Method by Principal Component Analysis Total Variance Explained for patients' data has been shown in Supporting Information S1: Table S2.

**Figure 5:**
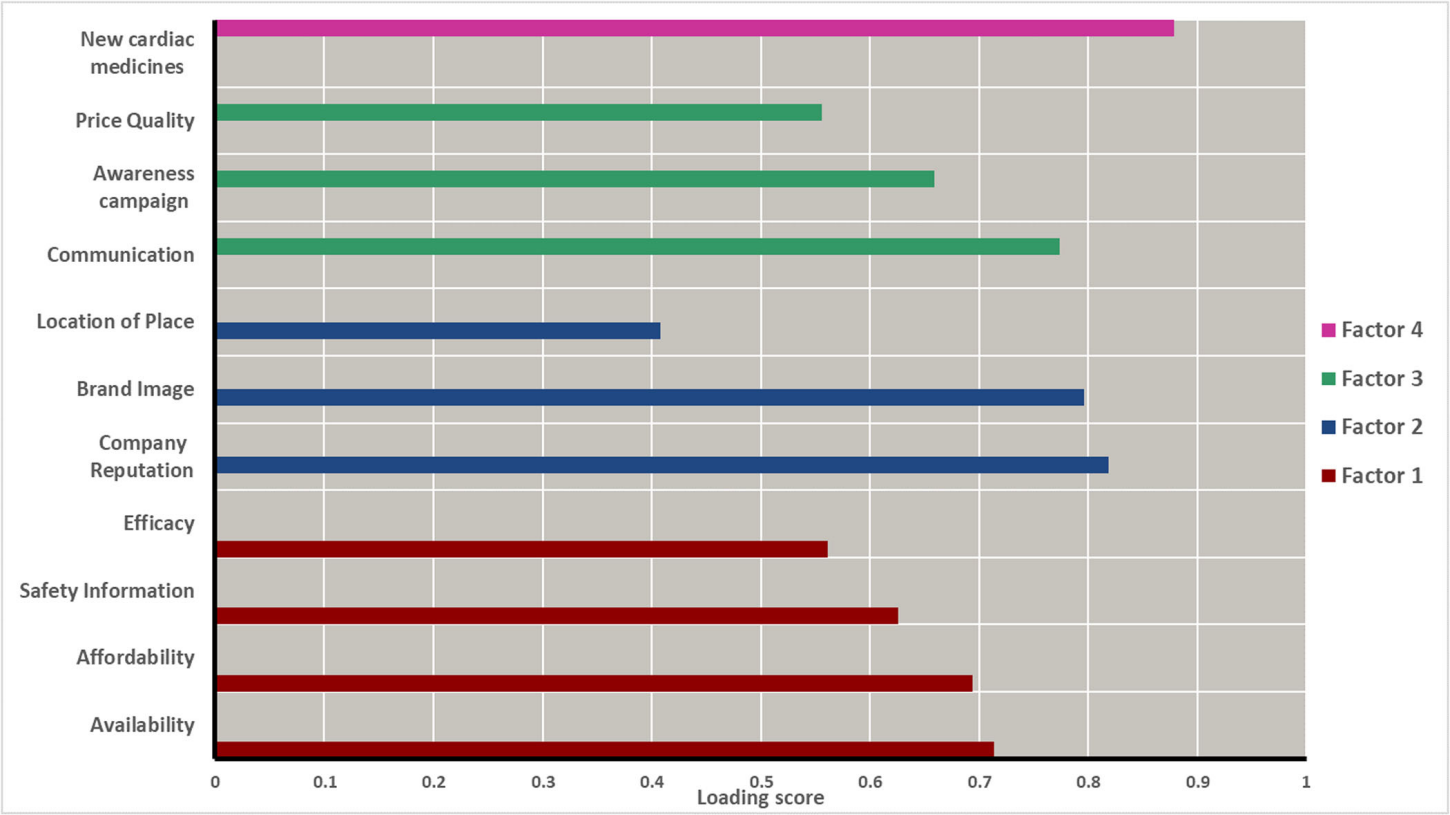
Bar plot showing factor loading scores of each variable affecting patient adherenece.

The following variables are grouped under Factor 1, which are availability, affordability, safety, and efficacy. Company reputation, brand image, and location of place were assorted under the category of Factor 2. Communication, awareness campaign, and price quality were grouped together under Factor 3. The variable assorted under Factor 4 was new cardiac medicines. In Figure 5, a bar plot was generated showing the loading scores of each variable assorted under the different factors.

### 3.7 Hypothesis Testing

Hypothesis Testing was carried out by Regression Analysis of Factors using data of patients (*n* = 200).

Patient satisfaction is positively impacted by medicine efficacy.

Patient satisfaction is positively impacted by the medicine's brand image.

Patients' satisfaction is positively impacted by safety information about medications.

Patient satisfaction is positively impacted by price signal quality.

Patient satisfaction is positively impacted by the availability of medications.

Patients' satisfaction is positively impacted by a pharmacy's location.

As summarized in Table 2, among the 6 hypotheses developed, 4 of these showed well accepted *p*‐values and thus the positive hypothesis was accepted and null hypothesis was rejected.

**Table 2:** Hypothesis testing by regression analysis of factors carried out using patients' data.

Research Hypotheses	Comment	*p*‐value	Factor Category	Factor Label
H_1_: Patient satisfaction is positively impacted by medicine efficacy.	Accepted	0.000	1	Economic—Medicinal benefit
H_5_: Patient satisfaction is positively impacted by the availability of medications	Accepted	0.000	1	Economic—Medicinal benefit
H_3_: Patients' satisfaction is positively impacted by safety information about medications	Accepted	0.000	1	Economic—Medicinal benefit
H_4_: Patient satisfaction is positively impacted by price signal quality.	Accepted	0.002	3	Medicinal—Communication benefit

## 4 Discussion

Coronary heart disease, rheumatic heart disease, stroke and other illnesses are among the category of heart and blood vessel disorders known as CVDs [25]. Heart attacks and strokes account for more than four out of every five CVD deaths, and 1 out of 3 of these deaths happen before the age of 70 [26, 27]. The importance of patient adherence to long‐term prescription drugs is paramount in the context of cardiovascular disease [28]. When using marketing representatives as respondents in Malaysia, researchers discovered the beneficial effects of product quality, medicine availability, branding awareness, packaging services, and distribution on market share [29]. In a recent study carried out in China, efficacy and safety of medicines were important determinants of essential medicine use as perceived by both prescribers and patients [30]. In the present study, the effect of important variables on patient satisfaction was evaluated from multiple dimensions.

Price is a measure of a product's value that reflects the equitable nature of the exchange of economic benefits between consumers and sellers [31]. If the pricing is chosen to maximize the seller's profits, the customer's purchase price may be unreasonably high [32]. 64.2% of patients disagreed that the quality of cardiac medication is not commensurate with its cost. 35.1% of those surveyed said they had no opinion about the price of drugs. From the perspective of the buyer, a product's pricing is a combination of the drug's quality and effectiveness, the prices offered by their competitors, and the individual financial benefits they anticipate from the purchase [33]. Each pharmaceutical product is created with the goal of successfully competing with the propositions of its rivals, which must contain benefits to both the product and the wider economy. Benefits from products or medications are care values [34]. These include effectiveness, safety, toleration, quality, and price [12].

Numerous factors, including product positioning, product distinctiveness, number of competitor companies, branding, substitute products, as well as the significance of products for treatment, influence the demand for medicines with elastic and inelastic pricing [11]. 51% of cardiac patients agreed with the assertion that medications availability in retail pharmacies impact their compliance. It was found that price is not the primary factor influencing purchase decisions. However, benefits of the product or perceived value of the medicine are important factors that may influence purchasing decisions [35].

Doctors prefer pharmaceutical firms that take into account patients' ability to pay to enhance their quality of life by lowering disease symptoms. Newer medications have better acceptability among patients if the efficacy is enhanced and patients are made aware of the effectiveness of new medicines over the older ones, according to 46% of doctors. While 6% disagreed, 93% of doctors believed that quality and efficacy were important factors in patient satisfaction. According to hypothesis testing, availability, safety information, communication with patient, price signal quality of cardiac medicine, awareness campaign to prevent cardiac disease and new cardiac medicine have positive impact on patients' satisfaction.

According to hypothesis testing, patient satisfaction is positively impacted by medicine efficacy. If the medicines the patients are using have positive health benefits, patients will be more likely to stick with treatment regimen. Also, if the prescribed medicines are available, patient satisfaction is positively impacted. Approximately 61% of respondents stated that patient satisfaction is impacted by safety information on the cardiac medications that the companies supply. Patients are influenced by the safety information given on medication label. The claim that the availability of drugs in retail pharmacies affects their compliance was accepted by 51% of cardiac patients. Pricing of good quality medicines have impact on patients' satisfaction. In the current research work, various factors were analysed from different perspectives. The perspectives of physicians, patients and manufacturers were incorporated to get a better view of the variables that may influence patient adherence to long‐term treatment regimen.

## 5 Conclusion

The morbidity and mortality rate of cardiovascular diseases are rising day‐by‐day. Drug adherence is an important aspect of successful treatment plan and positive outcome in long‐term chronic diseases. The findings of the current study are important indicators of factors that affect patient compliance and have an impact on patient adherence to long‐term prescription drugs with irreplaceable health benefits. Policy makers can focus on the most important factors and develop these in the context of cardiac medicine for the betterment of overall healthcare system in Bangladesh. Future studies should be undertaken for better comprehension of the variables that can directly influence patients' decision to comply with the prescribed treatment regimen for chronic diseases like cardiovascular disease.

## Author Contributions

**Jesmin Sultana:** conceptualization, methodology, writing – original draft, data curation. **Rumman Reza:** conceptualization, writing – original draft, methodology, visualization. **Mohammad Saifuddin:** software, data curation. **Zakir Hossain Bhuiyan:** conceptualization, writing – review and editing, supervision.

## Disclosure

All authors have read and approved the final version of the manuscript [CORRESPONDING AUTHOR or MANUSCRIPT GUARANTOR] had full access to all of the data in this study and takes complete responsibility for the integrity of the data and the accuracy of the data analysis.

## Conflicts of Interest

The authors declare no conflicts of interest.

## Transparency Statement

The lead author Jesmin Sultana affirms that this manuscript is an honest, accurate, and transparent account of the study being reported; that no important aspects of the study have been omitted; and that any discrepancies from the study as planned (and, if relevant, registered) have been explained.

## Supporting information

Supporting information.

## Data Availability

The data that supports the findings of this study are available in the supplementary material of this article.
