# Supplementary material for: Perception About Factors Affecting Patient Adherence With Cardiac Medicines: A Cross‐Sectional Study
Source: Health Sci Rep. 2025 Mar 5;8(3):e70532. doi: 10.1002/hsr2.70532 (PMC11882383; doi:10.1002/hsr2.70532)
Supplement: Supplementary file 1 — Supporting information. [file HSR2-8-e70532-s001.docx]

**Supplementary Materials**

**Table S1**: Communalities of each variable affecting patient adherence to cardiac medicine.

| Extraction Method: Principal Component Analysis. | | |
| --- | --- | --- |
| **Variables** | **Initial** | **Extraction** |
| Brand of cardiac medicine | 1.000 | 0.643 |
| Safety information of cardiac medicine | 1.000 | 0.571 |
| Price signal-quality of cardiac medicine | 1.000 | 0.439 |
| New cardiac medicines | 1.000 | 0.778 |
| Awareness campaign | 1.000 | 0.454 |
| Affordability of cardiac medicine | 1.000 | 0.548 |
| Availability of cardiac medicine | 1.000 | 0.638 |
| Efficacy of cardiac medicine | 1.000 | 0.430 |
| Communication with patients | 1.000 | 0.667 |
| Location/place of medicine purchase | 1.000 | 0.427 |

**Table S2**: Extracted Communalities from Extraction Method by Principal Component Analysis Total Variance Explained for patients’ data.

| Component | Initial Eigenvalues | | | Extraction Sums of Squared Loadings | | | Rotation Sums of Squared Loadings | | |
| --- | --- | --- | --- | --- | --- | --- | --- | --- | --- |
|  | Total | % of Variance | Cumulative % | Total | % of Variance | Cumulative % | Total | % of Variance | Cumulative % |
| 1 | 2.066 | 18.781 | 18.781 | 2.066 | 18.781 | 18.781 | 1.881 | 17.097 | 17.097 |
| 2 | 1.801 | 16.372 | 35.153 | 1.801 | 16.372 | 35.153 | 1.649 | 14.994 | 32.091 |
| 3 | 1.271 | 11.556 | 46.709 | 1.271 | 11.556 | 46.709 | 1.458 | 13.251 | 45.342 |
| 4 | 1.159 | 10.537 | 57.246 | 1.159 | 10.537 | 57.246 | 1.309 | 11.903 | 57.246 |
| Source: Prepared by Researcher **(**Extraction Method: Principal Component Analysis.) | | | | | | | | | |
